# Randomized trial of a portable HEPA air cleaner intervention to reduce asthma morbidity among Latino children in an agricultural community

**DOI:** 10.1186/s12940-021-00816-w

**Published:** 2022-01-03

**Authors:** Rebecca L. Drieling, Paul D. Sampson, Jennifer E. Krenz, Maria I. Tchong French, Karen L. Jansen, Anne E. Massey, Stephanie A. Farquhar, Esther Min, Adriana Perez, Anne M. Riederer, Elizabeth Torres, Lisa R. Younglove, Eugene Aisenberg, Syam S. Andra, Seunghee Kim-Schulze, Catherine J. Karr

**Affiliations:** 1 Department of Environmental & Occupational Health Sciences, University of Washington, 4225 Roosevelt Way NE, Suite 100, Seattle, WA 98105 USA; 2 Department of Statistics, University of Washington, Seattle, WA USA; 3 Department of Epidemiology, University of Washington, Seattle, WA USA; 4 Department of Health Services, University of Washington, Seattle, WA USA; 5 Yakima Valley Farm Workers Clinic, Toppenish, WA USA; 6 Northwest Communities Education Center, Radio KDNA, Granger, WA USA; 7 School of Social Work, University of Washington, Seattle, WA USA; 8 Department of Environmental Medicine & Public Health, Icahn School of Medicine at Mount Sinai, New York, NY USA; 9 Human Immune Monitoring Center, Department of Oncological Science, Icahn School of Medicine at Mount Sinai, New York, NY USA; 10 Department of Pediatrics, University of Washington, Seattle, WA USA

**Keywords:** Air pollution, Childhood asthma, HEPA air cleaner, Randomized controlled trial

## Abstract

**Background:**

Data on pediatric asthma morbidity and effective environmental interventions in U.S. agricultural settings are few. We evaluated the effectiveness of HEPA air cleaners on asthma morbidity among a cohort of rural Latino children.

**Methods:**

Seventy-five children with poorly controlled asthma and living in non-smoking homes were randomly assigned to asthma education alone or along with HEPA air cleaners placed in their sleeping area and home living room. The Asthma Control Test (ACT) score, asthma symptoms in prior 2 weeks, unplanned clinical utilization, creatinine-adjusted urinary leukotriene E4 (uLTE4 [ng/mg]), and additional secondary outcomes were evaluated at baseline, six, and 12 months. Group differences were assessed using multivariable-adjusted generalized estimating equations. Incident rate ratios of *ever* experiencing the metrics of poorer asthma health during follow-up (suboptimal asthma management) were estimated using Poisson regression models in secondary analysis.

**Results:**

Mean child age was 9.2 and 8.6 years in intervention and control groups, respectively, and two-thirds of participants were male. Primary analysis of repeated measures of ACT score did not differ between groups (HEPA group mean change compared to controls 10% [95% CI: − 12-39%]). A suggestion of greater decrease in uLTE4 (ng/mg creatinine) was observed (− 10% [95% CI: − 20 -1%]). Secondary analysis showed children with HEPAs were less likely to have an ACT score meeting a clinically defined cutoff for poorly controlled asthma using repeated measures (IRR: 0.45 [95% CI: 0.21–0.97]). In Poisson models, intervention participants had reduced risk of ever meeting this cutoff (IRR: 0.43 [95% CI: 0.21–0.89]), ever having symptoms in the past 2 weeks (IRR: 0.71 [95% CI: 0.52–0.98]), and lower risk of any unplanned clinical utilization (IRR: 0.35 [95% CI: 0.13–0.94]) compared to control participants.

**Discussion:**

The HAPI study showed generally improved outcomes among children in the HEPA air cleaner group. However, primary analyses did not meet statistical significance and many outcomes were subjective (self-report) in this unblinded study, so findings must be interpreted cautiously. HEPA air cleaners may provide additional benefit for child asthma health where traditional asthmagens (traffic, tobacco smoke) are not prominent factors, but larger studies with more statistical power and blinded designs are needed.

**Trial registration:**

ClinicalTrials.gov Identifier: NCT04919915. Date of retrospective registration: May 19, 2021.

**Supplementary Information:**

The online version contains supplementary material available at 10.1186/s12940-021-00816-w.

## Introduction

While the majority of research on pediatric asthma has focused on urban cohorts, the data on asthma prevalence and morbidity in rural USA settings suggest that prevalence and morbidity are as high or higher for minority and low-income rural children compared to their urban counterparts [1–4].

There is substantial evidence derived from urban settings that indoor and outdoor air quality influences pediatric asthma symptoms, lung function, and clinical utilization [5, 6]. In the agricultural Yakima Valley of Washington State, we conducted observational epidemiological studies that demonstrated associations between ambient particulate matter (PM_2.5_) and NH_3_ and asthma symptoms and lung function among children with asthma in this rural setting [7, 8]. Through an ongoing community partnership, we sought to conduct solution-oriented research to improve the health of children with asthma in this region.

We selected an indoor air cleaning intervention, recognizing that children spend substantial time indoors at home (70% for age 6–11 years) and outdoor pollutants of concern may infiltrate indoor environments [9, 10]. Furthermore, programs that include identification and control of household indoor triggers showed improved asthma outcomes among children with asthma in urban settings [11].

In recent years, a small number of interventions using high efficiency particulate air (HEPA) air cleaners to improve indoor air quality suggest that inclusion of HEPA air cleaners can have a positive effect on pediatric asthma symptoms and morbidity in non-agricultural settings impacted by traffic emissions, tobacco smoke, and industrial emissions [12–15]. One study examined effectiveness specifically during wintertime in rural homes using uncertified wood stoves [16]. While PM_2.5_ was reduced, the primary health outcome metric, asthma quality of life scores, were unchanged although diurnal variability in peak flow was reduced [16]. The effectiveness of air cleaners to reduce asthma morbidity has not been specifically examined among rural pediatric populations in agricultural settings.

The Home Air in Agriculture Pediatric Intervention (HAPI) Trial evaluated the effect of portable HEPA air cleaners on asthma morbidity among a cohort of rural Latino children participating in a community health worker (CHW) delivered asthma education program. The purpose of the study was to determine whether in-home HEPA air filtration in addition to asthma health education decreases asthma morbidity and asthma clinical utilization more than asthma education alone. We previously demonstrated that the HEPA air cleaners significantly reduced PM_2.5_ concentrations in children’s homes during the trial [17].

## Methods

As part of a community-engaged research partnership comprising investigators from the University of Washington (UW) Pacific Northwest Agricultural Safety and Health Center, the Yakima Valley Farm Workers Clinic (YVFWC), and the Northwest Communities Education Center/Radio KDNA, we conducted a randomized trial from July 2015 to February 2019 to assess the effectiveness HEPA air cleaners to improve the health of children with asthma. The study was conducted in the Lower Yakima Valley, a region largely comprised of immigrant Latino farm workers and their families who participate in the area’s intensive agricultural production. Pediatric asthma is a longstanding health concern in this community. The study protocol and methods are described in detail elsewhere [18].

### Recruitment and screening

From July 2015 to November 2017, participants were recruited from children referred to the YVFWC Asthma Education Program, an established CHW delivered program. YVFWC Asthma Education Program CHWs were trained and supervised by UW research staff to conduct screening and data collection for the HAPI Study. Children were eligible to participate if they met the following criteria: 1) aged 6–12 years; 2) live within 800 m of dairy or crop production; and 3) have poorly controlled asthma. The latter was defined as four or more days with asthma symptoms in the past 2 weeks, use of asthma rescue medications for four or more days in the past 2 weeks, hospitalization or emergency department visit for asthma in the past year, or unscheduled clinic visit due to an asthma attack in the past year. Potential participants were excluded if they lived with someone who smoked, had more than one primary residence, planned to leave Yakima for more than 2 months during the study intervention period, or planned to move within 6 months.

### Study enrollment and randomization

**Fig. 1:**
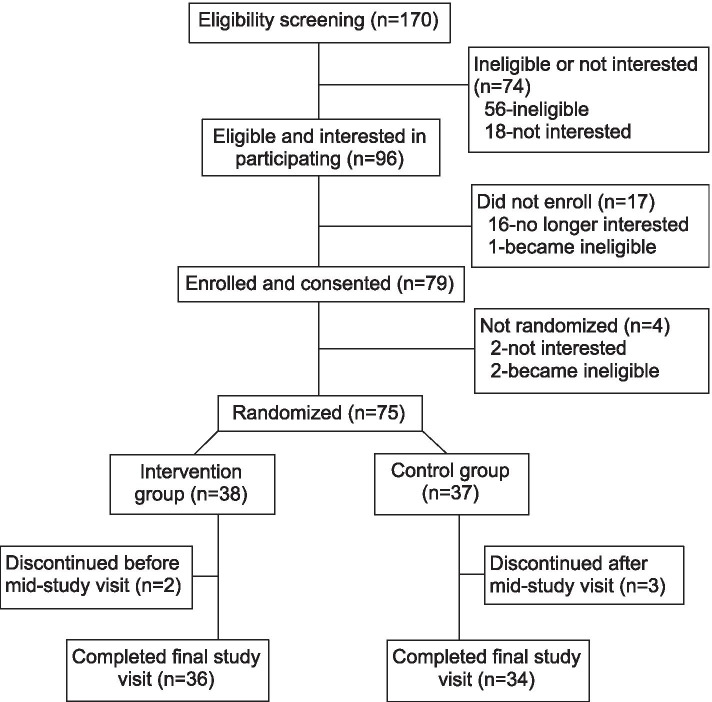
Participant screening, enrollment, randomization, and retention

Of 170 children who were screened, 96 (56%) were eligible (Fig. 1). Seventy-nine children (46%) followed up with an enrollment visit in clinic that included caregiver consent and child assent for study participation as well as the first session of the CHW standard three session asthma education that is available through the YVFWC for this community. The study baseline assessment in the participant’s homes was conducted on average 7-8 weeks later at which time participants were randomized for the trial. Participants were randomized to the intervention (HEPA air cleaner in addition to continuation of the standard asthma health education) or to the control group (continuation of the standard asthma health education alone). Participants were randomly assigned to the intervention group or the control group using sealed envelopes containing group assignment. Envelopes were prepared at the UW and unsealed by a UW study staff member just prior to the baseline assessment.

The UW Human Subjects Division and the YVFWC Research Review Committee approved the study. Participants who agreed to share clinical record data signed Health Insurance Portability and Accountability Act forms.

### Intervention

The intervention group received two portable Austin Air Pet Machine 410 HEPA Air Cleaners (Austin Air Systems Ltd., Buffalo, NY). One portable HEPA air cleaner was placed in the child’s sleeping area and a second air cleaner was placed in the home living room. Caregivers were instructed to keep the air cleaner running at the highest tolerable fan level at all times over the study year of follow-up and, if possible, to close the bedroom door. An Onset® HOBO UX90–004 Motor On/Off Data Logger (Onset Computer Corporation, Bourne, MA) that was designed to record the date/time with motor on or off events was placed in the sleeping area HEPA units and in half of the living HEPA units. Use of the air cleaners was also quantified subjectively via caregiver survey conducted at the mid-study and final study visit that recorded the reported use behavior regarding fan speed level used and the number of days each air cleaner was turned off in the prior 30 days. Intervention participants were allowed to keep the air cleaners after study completion. Control group participants were offered an air cleaner after completion of final data collection.

The three asthma education visits conducted by the YVFWC CHWs addressed proper medication technique, medication adherence, asthma trigger identification, and behavioral guidance on trigger control. As noted above, the first asthma education session occurred in the clinic at study enrollment. Importantly, this included review of the participants prescribed asthma medication management plan and steps to ensure access (e.g. refills, replacement) were taken if needed. The second asthma education session occurred during the home visit for the study’s baseline assessment. The last asthma education session was conducted in the home approximately 3 months after enrollment, independent of the study assessments. All participants also received dust mite covers for the child’s pillow and mattress, a cleaning kit with floor and surface cleaners free of irritant or toxic chemicals, a medication storage container, and a peak flow meter.

### Outcomes

Asthma-related health metrics were collected over the course of the participants’ year in the study collected during enrollment and trial data collection at baseline, mid-study, and end of study home visits. Metrics included clinically oriented measures related to asthma management (Asthma Control Test [ACT] score, days with asthma symptoms, urgent or unscheduled clinical utilization for asthma concerns, prescription for a course of oral steroids, and lung function. Biomarkers associated with asthma exacerbation, including fractional exhaled nitric oxide (FeNO) and urinary leukotriene E_4_ concentration (uLTE4) were also assessed.

We selected ACT scores, recent symptom days, unplanned clinical utilization, and uLTE4 (ng/mg creatinine or ng/mg) as primary outcomes. A power calculation estimated a repeat measure design would provide power to observe a 2-point change in the ACT score. We estimated to have adequate power to observe a two-point change in the raw ACT score. The ACT score is a validated composite metric to capture longer term asthma control (four-week referent period), the goal of asthma treatment. This is the one composite measure of asthma that has been identified as a recommended core metric for assessment of asthma control in NIH-initiated prospective trials in children less than age 12 years [19]. Symptom days in the past 2 weeks is a commonly assessed outcome in asthma intervention research and represents participant perception of more recent asthma-related disruption. Clinical utilization is an important measure for understanding individual and public health impacts of asthma morbidity and is arguably less subjective than caregiver or child report of asthma related symptoms and disruptions. Lastly, uLTE4 (ng/mg) was selected as a primary objective measure, because preliminary data in our research group had previously identified suggestive relationships between ambient sources of air pollution in this community and uLTE4 in children with asthma [20].

The ACT provides a composite score of disease activity based on a set of questions regarding symptoms during the most recent 4 weeks. This is a validated instrument available in English and Spanish [19]. Two versions of the ACT Questionnaire were used as appropriate for age: the five-item version for persons aged 12 or older and the seven-item Childhood ACT (C-ACT) version for children aged 4–11. Both questionnaires use 5-point and 6-point Likert scales with higher values indicating better asthma control with a highest possible score of 25 for the ACT and 27 for the C-ACT.

Caregivers were asked to report the total number of days with any asthma symptoms and to report the number of days with specific symptoms (wheezing, woken at night from asthma symptoms, stopped playing due to asthma symptoms, and days of missed school due to asthma symptoms) during the most recent 2 weeks. They were also asked to report number of unplanned or unscheduled visits to a clinic or emergency department for asthma symptoms and treatment.

For uLTE4 assessment, spot urine specimens were collected during each study visit. Aliquoted samples were stored at − 20 °C prior to submitting the urine samples to the National Institute of Health’s Children’s Health Exposure Analysis Resource for quantification of urinary creatinine using a G-EQUAS (http://www.g-equas.de/) proficiency test validated colorimetric method based on Taussky [21] and uLTE4 using an enzyme-linked immunosorbent assay (No. 501060; Cayman Chemical, Ann Arbor, Michigan). Creatinine-adjusted uLTE4 (ng/mg) was also calculated, using uLTE4 (ng/mL) divided by creatinine (mg/mL), for comparison with other studies. ULTE4 is an emerging biomarker in pediatric asthma research correlated with airway inflammation and asthma exacerbation [22].

Oral steroid prescriptions were identified by retrospective medical records review. The number of short-term steroid prescriptions was calculated using the total number of these prescriptions ordered at YVFWC clinics over a participant’s entire period of HAPI enrollment. Exhaled nitric oxide is an FDA approved approach for monitoring airway inflammation among individuals with asthma. Lung function was assessed with the EasyOne spirometer (NDD Technologies, Andover, MA) according to American Thoracic Society guidelines. Participant flow volume loops and volume time curves were reviewed and tests with evidence of cough or poor performance were not used. Participant sessions with an average of at least two exhalations with a forced exhaled volume (FEV) capacity difference of 0.15 L or less were used for analysis. FeNO was measured prior to spirometry via a portable NIOX VERO® device (Aerocrine Inc., Stockholm, Sweden and Aerocrine Corp.).

### Covariates

Socio-demographic, health, and psycho-social characteristics assessed by self-report on study surveys included: age, sex (male, female), Hispanic/Latino ethnicity, born in U. S, annual total household income (< $14,999, $15,000 – $29,999, $30,000 – $60,000, > $60,000), public health insurance, time spent in home in past 24 h (hours), home location (rural, on a farm; rural, not on a farm; in town), home within 800 m of pollution sources (farm raising animals, farm growing crops, roads with heavy traffic, unpaved dusty roads), and use of asthma controller medication (inhaled corticosteroid and/or leukotriene antagonist).

We assessed asthma-related stress using the caretaker’s response (strongly disagree, disagree, not sure, agree, strongly agree) to the following statement: My child’s asthma has caused stress in my family. A caretaker was categorized as having asthma-related stress if they answered “agree” or “strongly agree” to the statement. Child atopy was assessed at enrollment via skin prick test on the forearm using six common aeroallergens (cat, dog, mouse, mixed mold, dust mite, and cockroach). Participants were categorized as atopic if they had a wheal size equal to or greater than three millimeters above the negative control at 15 min after the test, a positive response to histamine, and a negative response to negative control.

At the enrollment visit research staff collected weight in kilograms using a Health-O-Meter scale (model 599KL) and collected height in centimeters using a single measurement with a wall-mounted stadiometer. Body mass index (BMI) was calculated as kilograms of weight divided by height in meters squared. Participants were categorized as overweight or obese if their BMI was at or above the 85th percentile for their age and sex [23, 24]. Height data after enrollment were attained through medical records review of all YVFWC visits during the HAPI enrollment period. We excluded YVFWC visit heights that were less than the HAPI enrollment height value and heights that were more than 25 percentiles different than the enrollment height-for-age percentile. We used each participant’s average age-for-height percentile from all heights after exclusions to estimate heights at the baseline, mid-study, and final study visits.

### Descriptive analysis

To examine the effectiveness of randomization, we assessed the comparability of demographic, psycho-social, and health-related characteristics of intervention and control group participants at randomization (study baseline) using chi-square test, analysis of variance, and Student’s t-test. Because all participants received an initial asthma health education session at enrollment, which addressed asthma symptom management and access to medications as prescribed, we also reviewed the health outcomes values at enrollment in addition to the trial baseline visit along with statistical comparison of the groups’ changes from enrollment to baseline.

Health outcomes were characterized using continuous and dichotomous values as follows. For the C-ACT and ACT questionnaires, participants were categorized as having poorly controlled asthma if they had a score of 19 or lower [25]. To evaluate absolute score differences from participants with adult (age > 12 year) and child versions of the ACT together, a standardized ACT score was created by dividing the participant’s total score by the total possible score to calculate a score percent. As noted above, the study was designed to detect a 2-point change in the raw ACT score. Of note, a 7.4-point change in the standardized ACT score is equivalent to a 2-point change in the raw score for the child version of the ACT and an 8-point change in the standardized ACT score is equivalent to a 2-point change in the raw score for the adult version of the ACT. Participants were categorized as having recent asthma symptoms if they answered “yes” to any of the asthma symptoms categories. Creatinine-adjusted uLTE4 was defined as high if it was at or above the baseline median value of 1.35 ng/mg. FeNO concentration was categorized as elevated if the FeNO value indicated an intermediate or high level of pulmonary inflammation according to American Thoracic Society guidelines [26]. For children less than 12 years old, FeNO was categorized as low (< 20 ppb), intermediate (20–35 ppb), or high (> 35 ppb) and older children were categorized as low (< 25 ppb), intermediate (25–50 ppb), or high (> 50 ppb). All spirometry results were interpreted using the multi-ethnic Global Lung Initiative reference eqs. [27]. FEV1 divided by forced vital capacity (FEV1/FVC) and FEV1 percent predicted value were calculated using age and estimated height on the date of spirometry. FEV1 percent predicted value was categorized as low if it was less than 80% of the predicted value as well as whether it was less than the lower limit of normal (LLN). LLN was defined as a z-score less than negative 1.645. FEV1/FVC was categorized as below normal if it was less than 0.80. Below normal forced expiratory flow (FEF) 25–75 was categorized as less than 60% of predicted value as well as whether it was below the lower limit of normal (LLN).

### Statistical analysis

For all outcomes with repeated measures (ACT, symptom days in prior 2 weeks, uLTE4, FeNO, and spirometry), we examined the difference between control and intervention participants for changes in these outcomes averaged over the mid-study and final visits using generalizing estimating equations (GEE) in linear models which account for within-subject correlation [28]. This GEE approach analyzing continuous outcome variables was considered the primary analytical approach. The study was designed to capture the mean outcome improvement over a year with repeat measures. Binomial, Gaussian, and Poisson versions of GEEs were used as appropriate for bivariate, continuous, and count variables. In secondary analysis GEE models examined dichotomous outcome variables for ACT using a clinically defined cutoff score, FeNO, symptom days, uLTE4, and spirometry as described above in the Descriptive Analysis section. GEE models included an interaction term for intervention with visit. Total number of unscheduled clinical utilization visits and steroid prescriptions over the study year were analyzed in linear regression models with robust standard errors. All models were adjusted for the baseline value of the outcome variable and adjusted for a priori determined covariates that are known to be strongly associated with pediatric asthma outcomes including sex, age, controller medication use, and season [29].

We also sought to characterize the effect of the intervention on achieving the overarching long-term goal of optimal asthma care, which is to achieve optimal asthma control, prevent symptoms, and enable patients to live without functional limitations or risk of adverse events (severe exacerbations, urgent clinical care). This was a secondary analytical strategy in which we categorized participants based on “ever” (suboptimal) versus “never” (optimal) having the poorer measure of asthma health status based on each dichotomous outcome after randomization. We estimated the incidence rate ratio (IRR) of suboptimal outcome during the year of follow-up for intervention children compared to control children using multivariable-adjusted Poisson regression models with robust standard errors adjusted for baseline value of the outcome metric, sex, age, controller medication use, and season. To reduce heteroscedasticity in the residual variance for the continuous outcomes standardized ACT score, FeNO and uLTE4 (ng/mg), we used the log-transformed values. To allow for log-transformed analysis for standardized ACT scores with a zero value the analysis used:

$$\mathit{\log}\left( standardized\ ACT\ score-\left(5/\left(100- standardized\ ACT\ score\right)-5\right)\right)$$

The percent of participant measurements unavailable for analysis did not significantly differ between intervention and control groups and was less than 7% for all primary outcome variables except for uLTE4 (ng/mg). At least one uLTE4 value was not included in the analysis for 35% of participants. This reflected agreements for the National Institutes of Health’s Children’s Health Exposure Analysis Resource laboratory processing of urine that were dependent on sending samples prior to completion of the final study visits for 12 participants. In addition, values were out of range for six participants, and samples were damaged or could not be collected for eight participants. Complete FeNO data was not available for analysis after enrollment for 13% of participants and steroid prescription and unplanned clinical utilization data over the study year was not available for 7 % of participants, due to loss to follow-up for five participants. At least one FEV1 value after enrollment was not included in the analysis for 43% of participants and at least one FEF25–75 and FVC values after enrollment was not included in the analysis for 53% of participants. Of the spirometry values not included in the statistical analysis, 34 participants had at least one value excluded due to inadequate quality and six participants were missing due to testing problems or missed visits. Spirometry is a supplemental analysis in this study given the small sample size. There was no missing data for the covariates used in the fully adjusted models. All statistical tests were 2-tailed and used a threshold of significance of *p* < 0.05. Statistical tests were conducted using Stata version 14.2 software (StataCorp, College Station, TX).

### Sensitivity analyses

In sensitivity analyses, first to understand the intervention effect among children with more severe asthma, we examined the subset of participants who used a controller medication at baseline (*N* = 67). Second, to investigate the role of intervention adherence, we conducted a sensitivity analysis in subset that excluded seven intervention participants who reported turning off the HEPA air cleaner more than 2 days during the month prior to their mid-study or final study visit. Next, we conducted a sensitivity analysis adjusting for nine study participants (6 intervention group participants and 3 control group participants) who also participated in a community-based home weatherization program that became available during the HAPI Study period, because in some cases the weatherization program included individualized components that overlapped with the HAPI study intent to reduce indoor asthma triggers. Components of the weatherization program including HEPA furnace filters and vacuum cleaners, air duct sealing, roof repair, exhaust fan replacement, and carpet replacement with laminated wood flooring. Lastly, we looked at effects on ACT score in the subset (*N* = 69) that completed the C-ACT at baseline, which did not require standardization for interpretation. Sensitivity analyses followed the same analytic models as the primary analysis except we did not examine spirometry outcomes in sensitivity analyses due to the small sample size.

## Results

On average, intervention participants were 9.2 (SD: 2.0) years old and control group participants were 8.6 (SD: 2.0) years old (Table 1). About one-third of participants in both groups were female. All participants were of Hispanic/Latino ethnicity and most were born in the USA. The total household income was less than $30,000 for 53% of the intervention group and 65% of the control group. All participants lived near farms growing crops and less than 50% lived near farms raising animals. At enrollment 55% of intervention group participants and 70% of control group participants had a BMI above normal and over 60% in each group were atopic. All assessed socio-demographic characteristics did not significantly differ between groups, consistent with successful randomization.

**Table 1:** Characteristics of HAPI Study participants

Characteristic	Intervention Group N (%)^**a**^	Control Group N (%)^**a**^	***p-value***
Number of participants	38 (100)	37 (100)	NA
Age, mean + SD, y	9.2 + 2.0	8.6 + 2.0	0.22
Female	14 (36.8)	13 (35.1)	0.88
Hispanic/Latino	38 (100)	37 (100)	1.00
Born in USA	38 (100)	35 (94.6)	0.15
Annual household income			0.14
< $14,999	2 (5.3)	9 (24.3)	
$15,000 – 29,999	18 (47.4)	15 (40.5)	
$30,000 – 60,000	15 (39.5)	11 (29.7)	
> $60,000	3 (7.9)	2 (5.4)	
Public health insurance	36 (94.7)	36 (97.3)	0.57
Hours spent inside home in past 24 h at baseline visit, mean + SD	16.5 + 4.7	17.0 + 3.3	0.64
Home location			0.67
Rural, on a farm	8 (21.1)	11 (29.7)	
Rural, not on a farm	10 (26.3)	8 (21.6)	
In town	20 (52.6)	18 (49.3)	
Home near (< 800 m) farm raising animals	15 (39.5)	19 (48.7)	0.42
Home near (< 800 m) farm growing crops	38 (100)	38 (100)	1.00
Home near (< 800 m) roads with heavy traffic	26 (68.4)	25 (67.6)	0.94
Home near (< 800 m) unpaved dusty roads	26 (68.4)	22 (59.5)	0.42
Season at baseline visit			0.75
Winter	9 (23.7)	13 (35.1)	
Spring	9 (23.7)	8 (21.6)	
Summer	10 (26.3)	8 (21.6)	
Autumn	10 (26.3)	8 (21.6)	
Body mass index (kg/m^2^) > 85th percentile	21 (55.3)	26 (70.3)	0.12
Prescribed controller medication	33 (86.8)	34 (91.9)	0.48
Atopy^b^	23 (60.5)	23 (62.2)	0.88
“My child’s asthma has caused stress in my family.” (strongly agree/agree)	14 (36.8)	17 (45.9)	0.42
“I am concerned about side-effects my child could get from taking asthma medicine for a long time.” (strongly agree/agree)	22 (57.9)	29 (78.4)	0.06

Notes: All characteristics collected prior to randomization

Footnotes: ^a^Presented as number and percent unless noted as mean and standard deviation

^b^Participants were categorized as atopic if they had at least one positive finding during skin prick testing for six common aeroallergens

The initial asthma health education session, which addressed medication use, access, and asthma trigger prevention, occurred after enrollment and prior to the study baseline visit. The improvement in health outcomes after this initial health education session (from enrollment to the baseline visit) did not significantly differ between groups for any of the primary outcomes (Supplemental Table 1), but it demonstrated substantial improvement prior to randomization. It is notable that 16% of the intervention group had an ACT score of 19 or less compared to 32% in the control group at baseline. However, the average standardized ACT test score was only 2% higher on average in the intervention group compared to the control group at baseline.

Of the 75 randomized participants, 73 (97%) completed the mid-study asthma assessment visit, and 70 (93%) completed the final asthma assessment (Fig. 1). All participants completed the three YVFWC asthma education sessions. Among intervention participants 81% reported turning off the HEPA air cleaners infrequently, on 3 days or less during the 30 days prior to the mid-study and final study visits. Over the course of the study year, on average both groups demonstrated modest improvement in almost all of the asthma outcomes based on repeated measures (Table 2). The mean standardized ACT score changed from 83.4 at baseline to a mean of 85.0 at the follow-up visits for the intervention participants. The mean standardized ACT score changed from 81.3 at baseline to a mean of 82.9 during the follow-up visits for the control group (Table 2). Analysis of changes in the primary outcomes with repeat measures using GEE (primary analytical strategy) showed that the mean change from baseline to follow-up was not significantly greater in the intervention group than in the control group (difference in mean change: 10% [95% CI: − 12-39%]). The change in uLTE4, the systemic biomarker of inflammation suggested greater reduction of this biomarker for intervention participants (difference in mean change: − 10% [95% CI: − 20- 1%]), but the confidence interval spanned the null.

**Table 2:** Asthma health outcomes at baseline and post-randomization visits and estimated effect of intervention on health outcomes

Clinical Outcome	Baseline Visit	6 and 12 Month Visits	Effect Estimate
Continuous Outcomes	Mean + SD	Mean + SD	Dif. in mean change (95% CI)
Standardized ACT score^c, d^			10% (− 12, 39%)
Intervention	83.4 + 9.53	85.0 + 9.63	
Control	81.3 + 11.3	82.9 + 12.2	
uLTE4 (ng/mg)^d^			−10% (−20, 1%)
Intervention	1.35 + 1.45	1.26 + 1.50	
Control	1.40 + 1.52	1.40 + 1.44	
FeNO (ppb)^d^			4% (−20, 34%)
Intervention	15.5 + 2.26	15.8 + 1.11	
Control	13.3 + 2.52	13.3 + 1.12	
			**Dif. in mean (95% CI)**
Unscheduled clinical utilization, visits			−0.14 (−0.48, 0.20)
Intervention	–	0.25 + 0.81	
Control	–	0.38 + 0.65	
Steroid prescriptions, n			−0.12 (− 0.70, 0.45)
Intervention	–	0.53 + 1.18	
Control	–	0.74 + 1.21	
**Dichotomous Outcomes**	**%**	**%**	**IRR (95% CI)**
C-ACT or ACT score < 19^a^, yes			0.45 (0.21, 0.97)
Intervention	15.8	8.3	
Control	32.4	20.0	
Symptoms in past 2 wk., yes			0.77 (0.52, 1.13)
Intervention	55.3	38.9	
Control	59.5	47.9	
ULTE_4_ (ng/mg) > median^b^, yes			0.77 (0.53, 1.11)
Intervention	46.0	37.5	
Control	54.3	51.6	
Elevated FeNO, yes			0.86 (0.49, 1.50)
Intervention	43.2	31.0	
Control	27.0	31.3	
**Discrete Outcome**	**Mean (SD)**	**Mean (SD)**	**IRR (95% CI)**
Symptoms in past 2 wk., days			0.63 (0.35, 1.11)
Intervention	1.63 + 1.88	1.43 + 2.53	
Control	2.03 + 2.90	2.04 + 3.54	

Notes: Effect estimates reflect comparison of the change from baseline to the mid-study (6 months) and final (12 months) visits averaged, in the intervention versus control groups; estimates were calculated using generalized estimating equations in repeated measures linear models that included an interaction term for intervention group and 6 and 12 month follow-up visit values and were adjusted for baseline outcome values, age, sex, season, and controller medication use for all outcomes except for unscheduled clinical utilization and steroid prescriptions. For unscheduled clinical utilization and steroid prescriptions, the difference between groups was estimated using multivariate adjusted linear regression models; C-*ACT* Childhood Asthma Control Test; *ACT* Asthma Control Test; *ULTE4* Creatinine-adjusted urinary leukotriene E4; *FeNO* Fractional exhaled nitric oxide

Footnotes: C-ACT or ACT score < 19 defines poorly controlled asthma

^b^UTLE4 median of 1.35 ng/mg

^c^Standardized ACT score is the total C-ACT or ACT score divided by the total score possible

^d^Standardized ACT score, ULTE4, and FeNO were log-transformed for analysis and, thus, the geometric mean for visits and the percent difference in mean change for the effect estimate are presented

In secondary analysis of primary outcome metrics using GEE models of dichotomous variables, the percent of participants who met the definition of poorly controlled asthma based on ACT score of 19 or less decreased from 15.8% at baseline to 8.3% during follow-up in the intervention group and from 32.4 to 20.0% in the control group (IRR: 0.45 [95% CI: 0.21–0.97], Table 2). For recent symptoms (prior 2 weeks), there was a greater reduction in the proportion of intervention group participants reporting any symptoms at follow-up compared with control group participants, but the differences were not statistically significant.

**Fig. 2:**
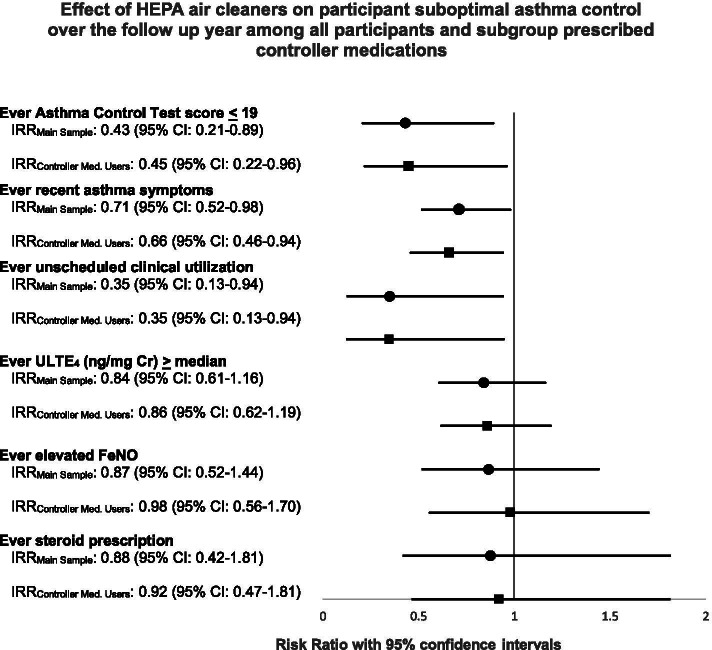
Intervention effect on *ever* having a suboptimal outcome during follow up based on asthma clinical measures of morbidity and biomarkers of inflammation estimated using Poisson regression models among all participants and among participants who used controller medications. Notes: IRRMain Sample: Model adjusted for age, sex, season at baseline, controller medication use, and baseline outcome value among main HAPI Study sample; IRRController Med. Users: Model adjusted for age, sex, season at baseline, and baseline outcome value among HAPI Study participants who used controller medication.

In secondary analysis using multivariable-adjusted Poisson regression models to examine the risk of *ever* experiencing the outcomes during the follow-up year, intervention group participants were less likely to have this indication of suboptimal asthma management based on several of the primary outcome measures (Fig. 2). Intervention children had reduced risk of ever ACT score of 19 or less (IRR: 0.43 [95% CI: 0.21–0.89]), reduced risk of ever asthma symptoms in the past 2 weeks (IRR: 0.71 [95% CI: 0.52–0.98]), and reduced risk of ever unplanned clinical utilization (IRR: 0.35 [95% CI: 0.13–0.94]) compared to control children (Fig. 2). The estimated risk of having uLTE4 (ng/mg) at or above the baseline median was lower in the intervention group than the control group, but the risk reduction estimate confidence interval included the null value (IRR: 0.84 [95% CI: 0.61–1.16]).

In analysis of secondary outcomes the mean number of steroid prescriptions was 0.53 in the intervention group and 0.74 in the control group (Table 2). The percent of participants with elevated FeNO decreased from 43.2% at baseline to 37.5% during follow-up in the intervention group and increased from 27.0 to 31.3% in the control group. These differences did not reach statistical significance in GEE repeated measure models nor in multivariable-adjusted Poisson regression analysis of ever elevated (Table 2, Fig. 2). In supplemental analysis of the reduced study sample with adequate spirometry results, there were no findings in repeated measures models for spirometry outcomes that supported improvement based on intervention status (Supplemental Table 2).

In the sensitivity analysis using multivariable-adjusted Poisson regression models limited to 67 participants who reported using controller medication at baseline, the intervention effect remained with similar reduction for the risk of ever having an ACT score of 19 or less (IRR: 0.45 [95% CI: 0.22–0.96], Fig. 2). The estimated rate ratio for ever experiencing recent asthma symptoms (IRR: 0.66 [95% CI: 0.46–0.94]) was estimated to show slightly more improvement comparing intervention to control, than for the main sample, while the risk of unscheduled clinical utilization for asthma (IRR: 0.35 [95% CI: 0.13–0.94]) was unchanged. In multivariable-adjusted repeated measures models among controller medication users the intervention effect reached statistical significance for recent asthma symptoms (IRR: 0.53 [95% CI: 0.29–0.98]). However, the estimated IRR confidence interval for ACT score representing poor control contained the null value (IRR 0.49 [95% CI: 0.23–1.05], Supplemental Table 3).

In the sensitivity analysis using multivariable-adjusted Poisson regression models limited to the intervention participants who reported that they did not turn off the HEPA air cleaner more than 3 days in the previous month (*N* = 68), we observed comparable risk estimates as observed in the full sample, with no changes in statistical significance. In multivariable-adjusted repeated measures models limited to this subset the intervention effect was now observed to be statistically significant for the number of recent days with asthma symptoms (Supplemental Table 4).

In the sensitivity analysis using multivariable-adjusted Poisson regression models that additionally adjusted for participation in the weatherization program, we observed no meaningful changes in results or interpretation of findings (Supplemental Fig. 2). In multivariable-adjusted repeated measures models adjusting for participants involvement in the weatherization program the intervention effect was also observed to be similar to effects observed in the main analysis (Supplemental Table 5).

In the sensitivity analysis of change in C-ACT score using multivariable-adjusted repeated measures models based on data from participants who completed the C-ACT for assessments (age < 12 years), the intervention effect was not statistically significant for an increase in C-ACT score in the main study sample or any of the three sensitivity analysis subgroups.

## Discussion

To our knowledge, this is the first HEPA randomized trial among children with asthma conducted in a rural, agricultural setting. We observed a suggested improvement for our primary measures of asthma health (ACT score, recent symptoms, unplanned clinical utilization) in the primary analysis that analyzed outcomes as continuous variables. However, these findings did not reach statistical significance. We observed statistically significant improvement in asthma outcomes for a one-year follow-up period among children provided portable HEPA air cleaners compared to children in the control group in secondary analysis of several of our measures of asthma health. Our most robust findings identified improvements in children meeting clinically defined asthma control, based on the Asthma Control Test, a validated composite metric used in both clinical settings and research. Our findings were robust in sensitivity analyses limited to children with more severe asthma, excluding less adherent participants, and adjusting for children who participated in a concurrent home weatherization program. Smoking prevalence is low in the study population of immigrant Latino families and the participating children with asthma in this study resided in non-smoking homes [30]. This study suggests that the effectiveness of this approach in improving pediatric asthma health extends to settings less influenced by the sources of pollution commonly addressed in asthma research (tobacco smoke, urban industrial or traffic emissions).

The study region, the Lower Yakima Valley of Washington State, experiences high ambient PM_2.5_ and has had longstanding community concerns about pediatric asthma morbidity. Ambient PM_2.5_ sources in the region include secondary aerosol formation from animal emissions, wood burning for heat, garbage or agricultural refuse burning, sporadic regional wildfire events as well as road and off-road vehicle emissions [18]. In our community-engaged research partnership, we previously demonstrated that use of HEPA air cleaners in this trial reduced geometric mean PM_2.5_ concentrations in the child’s sleeping area and home living room by an estimated 60 and 42% respectively, comparable to reductions observed in urban-based studies of HEPA air cleaner use [17]. The geometric mean indoor concentrations of PM_2.5_ measured at baseline were modest (11 μg/m3 for sleeping area, 12 μg/m^3^ for living room). While there are no widely accepted indoor air concentration guidelines, these levels approach current health-based regulatory thresholds for ambient air, such as the US Environmental Protection Agency National Ambient Air Quality Standard for annual average PM_2.5_ of 12 μg/m3. However, the levels are lower than those observed in USA homes with smokers. We hypothesized that HEPA air cleaners plus asthma health education sessions would decrease asthma morbidity more than asthma education alone for rural Latino farm worker children who have not been well-studied in prior asthma research.

To investigate this hypothesis, we examined multiple metrics to capture the influence of the intervention on asthma health. The overarching goal of asthma management is to minimize the disease impacts on the child. Children with optimally managed asthma are free of asthma symptoms and do not experience significant exacerbations or disruptions in their sleep and routine activities. This is challenging to achieve in some children as the factors influencing asthma control are many and may vary over time and a child’s environments. The ACT, one of our a priori selected primary outcomes, is a composite metric that provides a score based on a set of questions to characterize these components of asthma control in the past month. This represented a measure that best captured “longer term” control in our analysis. The ACT has been validated and used to identify asthma control based on an established score cut-off. It is also a measure that has been used by YVFWC’s asthma education program and has been recommended for use in asthma clinical trials [19]. Among our measured outcomes, we found the most robust evidence for our intervention in reducing morbidity based on ACT scores that represented poor asthma control based on a defined clinical cut off. We observed fewer children with poor asthma control in our HEPA group based on both repeated measures analysis to estimate mean effect over the year of follow up and in estimation of risk of ever experiencing poor asthma control comparing the groups.

While, in most cases, the changes for the rest of our primary outcomes (symptom reports, urinary leukotriene concentrations, and unscheduled clinical utilization) were more favorable for the HEPA group using our primary analytical strategy for repeat measure where available (Table 2), the estimates included confidence intervals spanning the null. A larger sample size may be required to confirm the influence of similar interventions on the selected outcome measures. Our study was powered to examine the ACT score as a continuous measure with repeat assessment (rather than defined based on meeting the clinical cutoff). We did not observe a significant effect on the continuous measure of ACT in the study which was estimated in the study proposal to have adequate power to observe a two-point change in the raw ACT score, which equates to a change of 8 or 7.4 for our standardized score for the childhood and adult versions of the ACT respectively.

In secondary analysis we characterized outcomes as suboptimal in regard to asthma management (i.e. ever experiencing the adverse outcome) and observed children in the HEPA group were more likely to maintain optimal disease management over the study year based on several of our metrics. Compared to control the group children, children in the HEPA group were estimated to have a 57% reduced risk of ever having poor asthma control based on composite ACT, a 28% reduction in risk of ever having days with asthma symptoms, and a 65% reduction in ever seeking urgent care for asthma problems. The results of our primary biomarker outcome of interest, uLTE4, were suggestive of a reduction among children in the HEPA group. This biomarker has not been used in asthma environmental intervention trials to date but has been of increasing interest as a biomarker of known mediators in airway inflammation and has been associated with asthma exacerbation [22]. Overall, these results are interpreted more cautiously as they were conducted as secondary analyses.

While several randomized trials have previously demonstrated the effectiveness of portable HEPA air cleaners to reduce indoor particulate matter in the homes of children with asthma, only a few have examined impacts on pediatric asthma morbidity. Unlike our study, prior studies almost exclusively address children residing in large urban settings [12–15, 31], although one focused on children in homes that relied on older woodstoves for heat in smaller sized towns in Montana, Idaho, and Alaska [16]. The outcome metrics assessed in published studies vary, making direct comparison with our results difficult. Like our study, most studies include some form of self-reported measures of asthma symptoms and many include measures of acute clinical utilization for asthma. A few report at least one spirometric measure [13, 15, 16] and one other reports exhaled nitric oxide levels [14]. Only one included a composite measure of asthma control [31] and none assessed uLTE4 concentration as a biomarker of inflammation.

The Inner City Asthma Study (ICAS) was a large, landmark multi-city trial that provided an individualized environmental intervention plan (based on individual child triggers) and portable HEPA air cleaners [15]. However, HEPA air cleaners were provided only for those children with exposure to tobacco smoke or sensitized and exposed to cat or dog and/or sensitized to mold. As such, the results cannot inform specifically on the effectiveness of the HEPA air cleaners. Notable findings included a reduction among the intervention group in the number of days with symptoms in the prior 2 weeks based on repeated measures over the intervention year and a decrease in asthma-related clinical utilization. No improvement in lung function was observed. In a somewhat similar design to the ICAS and our study, Eggleston et al. [13] recruited children who had completed an asthma education program and randomized participants to receipt of a HEPA air cleaner for the child’s bedroom, pest control for cockroach and rodent problems if present, and mattress and pillow covers. During the one-year follow-up period, the prevalence of daytime symptoms was reduced but not statistically significant in repeated measures analysis (OR: 0.62 [95% CI: 0.36–1.05]). This was somewhat comparable to our estimate of the IRR for symptoms in the past 2 weeks based on repeated measures over 1 year of follow-up (IRR: 0.77 [95% CI: 0.52, 1.13]). Nighttime symptoms, clinical utilization for acute asthma visits, and spirometry were also examined and not observed to be different from controls. Lanphear et al.’s [14] trial of active HEPA vs sham HEPA filters in homes of children with smokers observed a statistically significant reduction in acute asthma care visits over a year of follow-up for those equipped with active filter HEPA air cleaners. However, as in our study, FeNO levels, and prescriptions for steroids were not significantly reduced significantly in the intervention group. The proportion of days with symptoms in the prior 2 weeks did not vary among intervention participants versus controls.

In Butz et al. [12], which also recruited children in homes with smokers, two interventions (HEPA plus health coach to reduce smoking or HEPA alone) were compared to a control group. As in our study, all groups (including controls) received a set of general asthma education sessions. Over the study six-month follow-up, children in homes that were provided HEPA air cleaners (both intervention groups) demonstrated fewer days with symptoms (reported as symptom free days) compared to children in the control group. However, significant reduction in nighttime symptoms, slowed activity days, or acute care visits for asthma were not observed in the intervention group compared to the control group. Noonan et al. [16] examined the impacts of two interventions (improved Environmental Protection Agency-certified woodstove change out, HEPA air cleaner in the room with woodstove) compared to a sham HEPA air cleaner [16]. The primary health outcome metric, pediatric asthma quality of life score, was not improved among intervention participants. Secondary outcomes, FEV1 percent predicted value and peak expiratory flow rate were also not significantly different, however, the diurnal variability in peak expiratory flow rate were reduced (i.e., improved) among intervention participants.

Lastly, the most recent published trial examined the effectiveness of HEPA air cleaners using a crossover design, where individuals served as their own controls [31]. Participants included children impacted by traffic emissions (estimated using proximity-based exposure mapping). A HEPA air cleaner or sham HEPA was placed in the home for 4 weeks, followed by a one-month washout, and then participants crossed over to the other treatment arm for 4 weeks. Asthma control was assessed using the Asthma Control Questionnaire and asthma quality of life was assessed using the asthma Quality of Life Questionnaire. Statistically significant improvements were observed for the intervention period in the subgroup analysis of participants who met the definition of poor asthma control and quality of life at baseline.

Our study had several limitations to consider in interpretation of findings. Firstly, our participants were not blinded to their assignment. When developing the study, most comparable published intervention trials to improve pediatric asthma by reducing small particles using HEPA air filters had been unblinded [13, 15, 32], although Lanphear et al. 2011 had reported using sham filtration in a study focused on children in smoking homes in Cincinnati [14]. We discussed sham filter use with our community study partners during the design phase and the team decided use of sham would cause distrust and hinder participation. Community engaged research practice requires the trust and buy in of community partners, particularly in settings with immigrant and undocumented individuals. The lack of blinding introduces the potential for reporting bias for subjective outcomes in our study including ACT score and symptom days in prior week. We also examined unscheduled clinical utilization and a parent seeking clinical utilization in an urgent care clinic or emergency department, which may be a less subjective outcome than self-reported and perceived symptoms. Another limitation was the unexpected large improvement in asthma health outcomes (notably ACT and symptom days) observed in our participants prior to randomization, and in relation to a pre-study asthma education visit. This sizable improvement from the first education visit prior to the intervention start reduced our estimated power to see an intervention effect particularly for symptom days and ACT outcomes. This large asthma education effect was not anticipated or considered in the proposal power calculation.

We also experienced challenges in spirometry data quality based on household assessed lung function by CHWs at follow-up. Data loss due to poor participant performance in the spirometric maneuver and reliance on medical chart data to estimate height at follow-up assessment decreased power (sample size) and likely introduced noise, respectively. This limits the robustness of the findings, although as described above, the few prior HEPA trials for children with asthma that have included spirometric measures have generally not found significant influences on measures of lung function in children in this age group [13–16]. We also had a reduced number of the final study visit uLTE4 measurements as an artifactual feature of our arrangement with the National Institutes of Health’s Children’s Health Exposure Analysis Resource laboratory, which reduced our study power for this outcome. The study community is recognized by public health agencies for vulnerabilities based on socioeconomic factors and environmental concerns and an additional resource, the home weatherization program, became available to community members during our study. Eleven HAPI participants concurrently participated in the HAPI Study and the weatherization program, which provided participants with materials that may have changed indoor air quality. The potential for this to influence our findings was examined and no meaningful influence on primary analysis results was observed. Lastly, while we designed the study to collect self-reported and objective measures of adherence to the HEPA air cleaners in intervention households, the HOBO devices installed on the HEPA air cleaners to objectively determine on/off status failed in the majority of cases [17]. Thus, we were limited to the self-reported data on HEPA air cleaner use to understand intervention adherence. Prior studies have observed that poor compliance with recommended HEPA air cleaner use can be important to asthma outcomes [32] and low compliance would be expected to bias our results toward null effects.

The study had several strengths that support its contribution to a scant literature on interventional approaches to improve asthma, particularly in vulnerable, hard to reach communities historically underserved in asthma research. Retention was strong, likely attributable to the community-engaged nature of this study which involved trusted community partners in its design, conduct, and participant contact. We were able to characterize several features of asthma health, including an emerging biomarker, uLTE4, while maintaining a priori primary outcomes to reduce overinterpretation in a setting of multiple testing. The outcome metrics capture unique components of asthma morbidity of interest to clinicians and public health decision-makers. A key contribution of this particular study design was to capture the added value of HEPA air cleaners, above and beyond the recognized standard of care, which includes education addressing proper medication management, recognition and appropriate response to early signs and symptoms, and identification and reduction of common triggers [33]. Study partner YVFWC has experience providing a longstanding CHW-delivered asthma education program in this community and was vital to the study success. Asthma education delivered in the home by CHWs, has been established to be effective in improving pediatric asthma outcomes [34]. All participants also received low cost durable goods that are commonly part of asthma education programs for allergen and irritant reduction (i.e., dust mite pillows, mattress covers, green cleaning agents). At the completion of the trial, control participants were offered HEPA air cleaners and all but one family requested one.

## Conclusions

Asthma in children remains a major public health problem with significant disparities identified by sociodemographic risk factors and environmental conditions. The need for research to shift to interventional approaches is high. The influence of particulate matter as an asthma trigger is now well-established, as is the effectiveness of HEPA air cleaners to reduce indoor particulate matter. This study extends a growing evidence base demonstrating that HEPA air cleaner use in the home is an acceptable and important tool to consider in optimizing asthma control in children. Future research exploring this topic will benefit from selecting recommended outcome metrics [35], ensuring adequate sample size, focusing on vulnerable populations that may not have been served in prior research, and using protocols with blinding techniques acceptable to participants, including sham filters or cross over designs. Based on the evidence to date, for low-income children in settings where PM sources are a concern, instituting reimbursement policies for HEPA air cleaner prescriptions would provide a readily available component to complement general asthma education and medication management. Together, these strategies hold promise in achieving the goal of child days without symptoms, without disruption of sleep or desired activities, and without unnecessary clinical utilization.

## Supplementary Information

**Additional file 1.**

**Additional file 2.**

## Data Availability

The datasets used and analyzed during the current study are available from the corresponding author on reasonable request.

## Abbreviations

ACT: Asthma Control Test
BMI: Body mass index
C-ACT: Childhood Asthma Control Test
CHW: Community health worker
FEF: Forced expiratory flow
FeNO: Fractional exhaled nitric oxide
FEV: Forced exhaled volume
FVC: Forced vital capacity
GEE: Generalizing estimating equations
HAPI: Home Air in Agriculture Pediatric Intervention
HEPA: High efficiency particulate air
IRR: Incidence rate ratio
LLN: Lower limit of normal
PM2.5: Particulate matter less than 2.5 microns in diameter
uLTE4: Creatinine-adjusted urinary leukotriene E4
UW: University of Washington
YVFWC: Yakima Valley Farm Workers Clinic
