# Supplementary material for: Randomized trial of a portable HEPA air cleaner intervention to reduce asthma morbidity among Latino children in an agricultural community
Source: Environ Health. 2022 Jan 3;21:1. doi: 10.1186/s12940-021-00816-w (PMC8722199; doi:10.1186/s12940-021-00816-w)
Supplement: Supplementary file 1 — Additional file 1. [file 12940_2021_816_MOESM1_ESM.pdf]

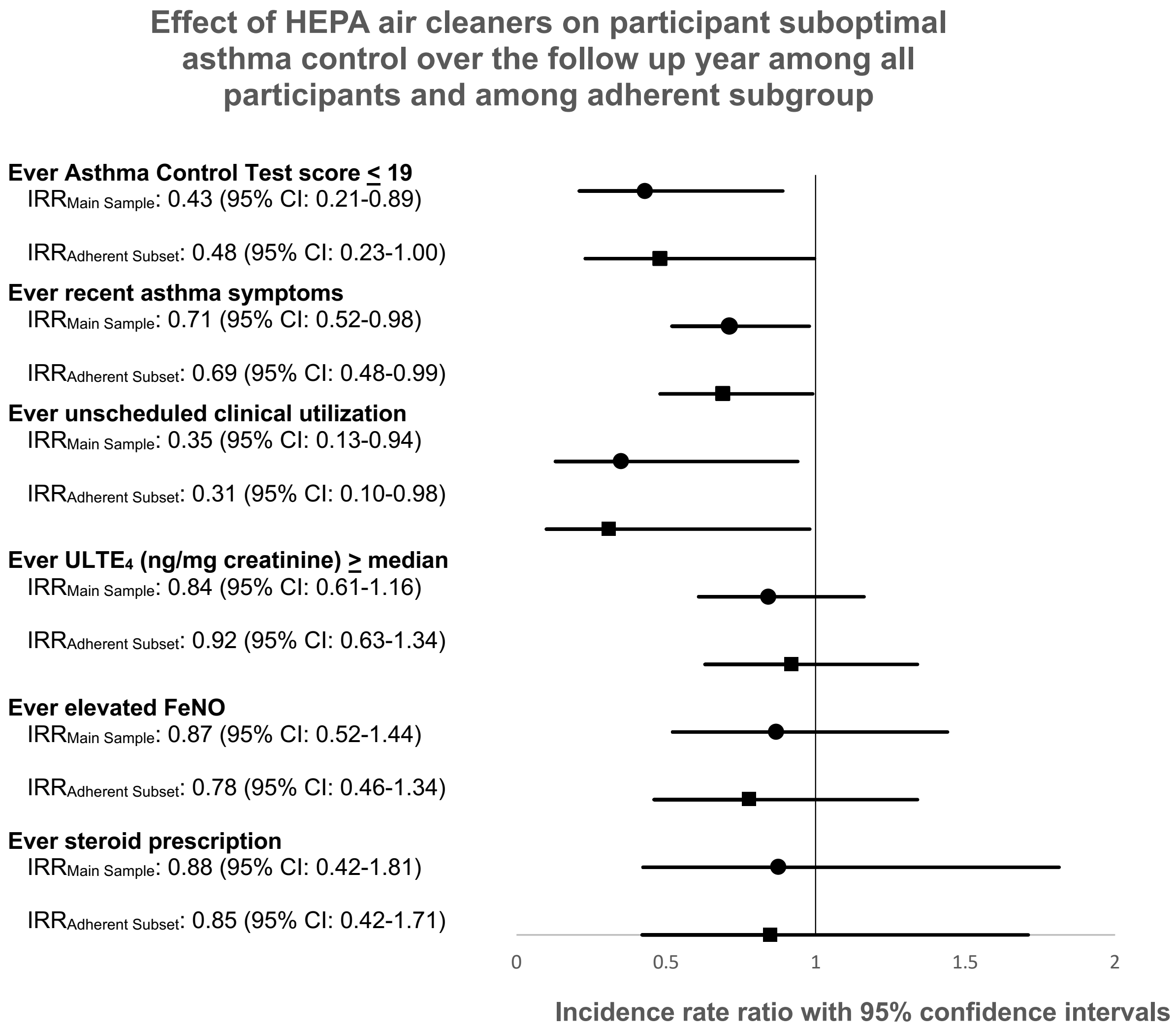

Supplemental Figure 1: Intervention effect on *ever* having a suboptimal outcome during follow up based on asthma clinical measures of morbidity and biomarkers of inflammation estimated using Poisson regression models among all participants and among adherent subgroup.

Notes: $IRR_{Main\ Sample}$: Model adjusted for age, sex, season, controller medication use, and baseline outcome value among main HAPI Study sample; $IRR_{Adherent\ Subset}$: Model adjusted for age, sex, season, controller medication use, and baseline outcome value among HAPI Study participants excluding 7 nonadherent intervention participants.

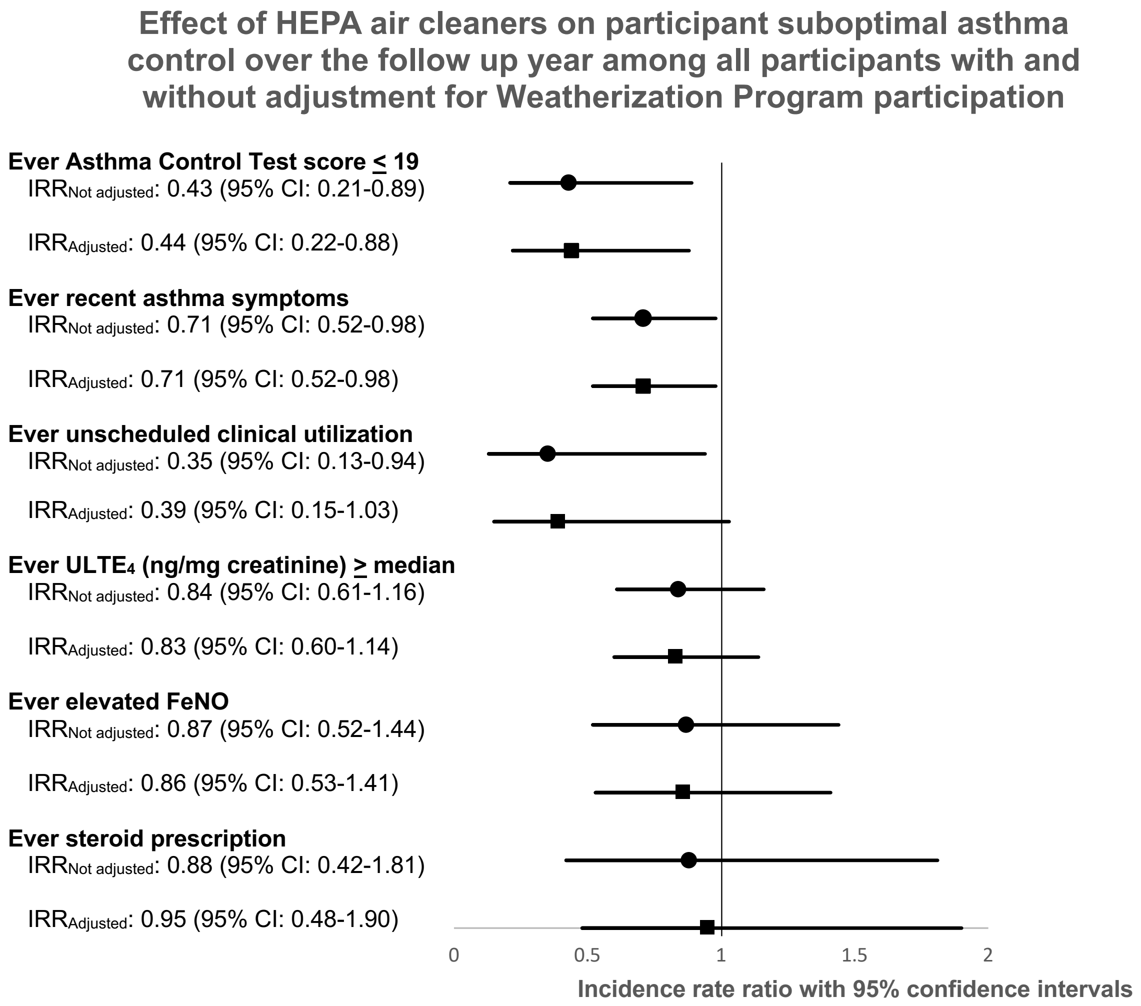

Supplemental Figure 2: Intervention effect on *ever* having a suboptimal outcome during follow up based on asthma clinical measures of morbidity and biomarkers of inflammation estimated using Poisson regression models among all participants with and without adjustment for Weatherization Program.

Notes: $IRR_{Not\ adjusted}$: Model adjusted for age, sex, season at baseline, controller medication use, and baseline outcome value, but not adjusted for Weatherization Program participation; $IRR_{Adjusted}$: Model adjusted for age, sex, season at baseline, baseline outcome value, and additionally adjusted for Weatherization Program participation.
