# Supplementary material for: Randomized trial of a portable HEPA air cleaner intervention to reduce asthma morbidity among Latino children in an agricultural community
Source: Environ Health. 2022 Jan 3;21:1. doi: 10.1186/s12940-021-00816-w (PMC8722199; doi:10.1186/s12940-021-00816-w)
Supplement: Supplementary file 2 — Additional file 2. [file 12940_2021_816_MOESM2_ESM.docx]

| **Supplemental Table 1: Enrollment and baseline measures of asthma health comparing change between HAPI Study intervention and control groups before and after asthma health education session.** | | | | | | |
| --- | --- | --- | --- | --- | --- | --- |
|  | **Enrollment Visit**  **(Before 1^st^ Asthma Education Session)** | |  | **Baseline Visit**  **(After 1^st^ Asthma Education Session)** | |  |
| **Outcomes** | **Intervention Group**  **N (%)^a^** | **Control**  **Group**  **N (%)^a^** |  | **Intervention Group**  **N (%)^a^** | **Control**  **Group**  **N (%)^a^** | ***p-value*** |
| **Clinical Outcomes** |  |  |  |  |  |  |
| C-ACT or ACT score < 19^b^ | 21 (55.3) | 16 (44.4) |  | 6 (15.8) | 12 (32.4) | 0.10 |
| Standardized ACT score, mean + SD^c, d^ | 71.3 + 13.9 | 72.9 + 14.4 |  | 83.4 + 9.5 | 81.3 + 11.3 | 0.43 |
| Symptoms past 2 wk, yes | 25 (65.8) | 26 (70.3) |  | 21 (55.3) | 22 (59.5) | 0.92 |
| Daytime wheeze or cough | 22 (57.9) | 24 (48.7) |  | 17 (44.7) | 18 (48.6) | 0.92 |
| Woken at night from symptoms | 14 (36.8) | 13 (35.1) |  | 7 (18.4) | 9 (24.3) | 0.06 |
| Stopped playing due to symptoms | 16 (42.1) | 18 (48.6) |  | 8 (21.1) | 5 (13.5) | 0.27 |
| Missed school due to symptoms^e^ | 5 (16.1) | 7 (21.9) |  | 2 (6.5) | 4 (12.5) | 0.99 |
| Symptoms in past 2 wk, mean + SD, d | 2.2 + 2.7 | 4.0 + 4.6***** |  | 1.6 + 1.9 | 2.0 + 2.9 | 0.59 |
| Daytime wheeze or cough, mean + SD, d | 2.1 + 2.7 | 3.8 + 4.4 |  | 1.2 + 1.7 | 1.9 + 3.0 | 0.22 |
| Woken at night, mean + SD, d | 1.2 + 2.6 | 2.1 + 4.0 |  | 0.4 + 1.0 | 0.7 + 1.4 | 0.38 |
| Stopped play, mean + SD, d | 1.6 + 3.3 | 2.6 + 4.0 |  | 0.6 + 1.5 | 0.6 + 1.7 | 0.76 |
| Missed school, mean + SD, % of d^e^ | 3.2 + 7.4 | 5.5 + 13.8 |  | 1.0 + 4.0 | 2.2 + 6.6 | 0.12 |
| **Biomarkers of Inflammatory Response** |  |  |  |  |  |  |
| ULTE_4_ (ng/mg), mean + SD^d^ | 1.24 + 1.38 | 1.37 + 1.41 |  | 1.35 + 1.45 | 1.40 + 1.52 | 0.50 |
| Elevated FeNO^d, f^ | 14 (43.2) | 9 (24.3) |  | 16 (43.2) | 10 (27.0) | 0.14 |
| FeNO (ppb,) mean + SD^d^ | 16.5 + 2.6 | 13.9 + 2.9 |  | 15.5 + 2.3 | 13.3 + 2.5 | 0.80 |
| **Spirometry** |  |  |  |  |  |  |
| FEV1% < 80% of predicted value | 4 (11.8) | 3 (10.7) |  | 8 (24.2) | 1 (3.5)* | 0.19 |
| FEV1 PP, mean + SD | 92.7 + 13.7 | 97.6 + 15.6 |  | 92.3 + 12.7 | 98.5 + 11.4 | 0.06 |
| FEF 25-75 < 60% of PP value | 11 (37.9) | 5 (20.0) |  | 10 (31.3) | 4 (15.4) | 0.23 |
| FEF 25-75% PP, mean + SD | 70.5 + 25.2 | 80.5 + 29.4 |  | 73.9 + 24.6 | 85.2 + 28.6 | 0.08 |
| FEV1/FVC ratio < 80% | 18 (62.1) | 11 (44.0) |  | 15 (46.9) | 10 (38.5) | 0.51 |
| FEV1/FVC ratio, mean + SD | 0.78 + 0.07 | 0.8 + 0.09 |  | 0.80 + 0.08 | 0.8 + 0.07 | 0.37 |
| FEV1% < LLN | 5 (14.7) | 3 (10.7) |  | 8 (24.2) | 1 (3.5)* | 0.19 |
| FEV1 z-score, mean + SD | -0.62 + 1.17 | -0.20 + 1.31 |  | -0.64 + 1.08 | -0.12 + 0.94 | 0.06 |
| FEF 25-75 < LLN | 12 (41.4) | 6 (24.0) |  | 14 (43.8) | 4 (15.4)* | 0.05 |
| FEF 25-75% z-score, mean + SD | -1.42 + 1.22 | -0.9 + 1.41 |  | -1.25 + 1.19 | -0.70 + 1.28 | 0.07 |
| FEV1/FVC < LLN | 16 (55.2) | 8 (32.0) |  | 11 (34.4) | 7 (26.9) | 0.72 |
| FEV1/FVC z-score, mean + SD | -1.48 + 0.95 | -1.06 + 1.21 |  | -1.15 + 1.06 | -0.9 + 1.08 | 0.36 |
| Notes: P-values presented are from comparing the difference in change between groups from enrollment (before asthma health education) to the baseline visit calculated using regression models adjusted for age, sex, season, controller medication use, and enrollment outcome value; Differences between groups at enrollment and at baseline within also separately calculated with using chi-square test and ANOVA and significant differences are noted with an asterisk in the cell; LLN: Lower limit of normal (z-score < -1.645); C-ACT: Childhood Asthma Control Test; ACT: Asthma Control Test; ULTE4: Creatinine-adjusted urinary leukotriene E4; FeNO: Fractional exhaled nitric oxide; FEV: Forced exhaled volume; FEF: Forced expiratory flow; PP: Percent predicted.  Footnotes:^a^Presented as number and percent unless noted as mean and standard deviation;  ^b^C-ACT or ACT score < 19 indicates poorly controlled asthma;  ^c^Standardized ACT score is the total C-ACT or ACT score divided by the total score possible;  ^d^Standardized ACT score, ULTE4, and FeNO were log-transformed for analysis and, thus, the geometric mean is presented.  ^e^Missed school days among 63 participants who reported school was in session;  ^f^Elevated fractional exhaled nitric oxide (FeNO) concentration categorized as elevated > 20 ppb for children < 12 years old and > 25 ppb for children > 12 years old. | | | | | | |

| **Supplemental Table 2: Spirometry health outcomes at baseline and post-randomization visits and effect of intervention on health outcomes post-randomization comparing intervention to control groups.** | | | | | |
| --- | --- | --- | --- | --- | --- |
| **Spirometry Outcomes** | **Baseline**  **Visit** |  | **Mean of 6 and 12 Month Visits** | | **Effect Estimate** |
| **Continuous Outcomes** | **Mean + SD** |  | **Mean + SD** | | **Dif. in mean change** |
| FEV1 PPV, mean |  |  |  |  | -1.46 (-5.87, 2.96) |
| Intervention group | 92.3 + 12.7 |  | 89.4 + 14.0 | |  |
| Control group | 98.5 + 11.4 |  | 96.1 + 13.8 | |  |
| FEF 25-75% PP, mean |  |  |  |  | 0.97 (-5.98, 7.92) |
| Intervention group | 73.9 + 24.6 |  | 73.5 + 23.1 | |  |
| Control group | 85.2 + 28.6 |  | 82.6 + 26.0 | |  |
| FEV1/FVC ratio, mean |  |  |  |  | 0.01 (-0.02, 0.04) |
| Intervention group | 0.80 + 0.08 |  | 0.81 + 0.08 | |  |
| Control group | 0.82 + 0.07 |  | 0.82 + 0.09 | |  |
| FEV1% z-score, mean |  |  |  |  | -0.13 (-0.50, 0.24) |
| Intervention group | -0.64 + 1.08 |  | -0.88 + 1.18 | |  |
| Control group | -0.12 + 0.94 |  | -0.33 + 1.15 | |  |
| FEF 25-75% z-score, mean |  |  |  |  | 0.05 (-0.30, 0.40) |
| Intervention group | -1.25 + 1.19 |  | -1.26 + 1.13 | |  |
| Control group | -0.70 + 1.28 |  | -0.84 + 1.26 | |  |
| FEV1/FVC z-score, mean |  |  |  |  | 0.15 (-0.23, 0.53) |
| Intervention group | -1.15 + 1.06 |  | -0.99 + 1.06 | |  |
| Control group | -0.87 + 1.08 |  | -0.76 + 1.21 | |  |
| **Dichotomous Outcomes** | **%** |  | **%** | | **IRR (95% CI)** |
| FEV1% < 80% of PV, % |  |  |  |  | 1.16 (0.52, 2.57) |
| Intervention group | 24.2 |  | 23.0 | |  |
| Control group | 3.5 |  | 10.2 | |  |
| FEF 25-75 < 60% of PPV, % |  |  |  |  | 0.90 (0.47, 1.75) |
| Intervention group | 31.3 |  | 24.6 | |  |
| Control group | 15.4 |  | 18.9 | |  |
| FEV1/FVC ratio < 0.80, % |  |  |  |  | 0.64 (0.38, 1.10) |
| Intervention group | 46.9 |  | 29.8 | |  |
| Control group | 38.5 |  | 32.1 | |  |
| FEV1% < LLN, % |  |  |  |  | 1.33 (0.51, 3.44) |
| Intervention group | 24.2 |  | 3.0 | |  |
| Control group | 3.5 |  | 8.5 | |  |
| FEF 25-75 < LLN, % |  |  |  |  | 0.96 (0.52, 1.78) |
| Intervention group | 43.8 |  | 29.8 | |  |
| Control group | 15.4 |  | 20.8 | |  |
| FEV1/FVC < LLN, % |  |  |  |  | 0.71 (0.39, 1.30) |
| Intervention group | 34.4 |  | 26.3 | |  |
| Control group | 26.9 |  | 24.5 | |  |
| Notes: Effect estimates reflect comparison of the change from baseline to the mid-study (6 months) and final (12 months) visits averaged, in the intervention versus control groups; estimates were calculated using generalized estimating equations in repeated measures linear models that included an interaction term for intervention group and 6 and 12 month follow-up visit values and were adjusted for baseline outcome values, age, sex, season, and controller medication use for all outcomes; LLN: Lower limit of normal (z-score < -1.645); FEV: Forced exhaled volume; FEF: Forced expiratory flow. | | | | | |

| **Supplemental Table 3: Asthma health outcomes at baseline and post-randomization visits and effect of intervention on health outcomes post-randomization comparing intervention to control groups among 67 HAPI Study participants using controller medication.** | | | | |
| --- | --- | --- | --- | --- |
| **Clinical Outcome** | **Baseline**  **Visit** |  | **6 and 12 Month Visits** | **Effect Estimate** |
| **Continuous Outcomes** | **Mean + SD** |  | **Mean +** **SD** | **Dif. in mean change (95% CI)** |
| Standardized ACT score^c, d^ |  |  |  | 9% (-14%, 40%) |
| Intervention | 83.1 + 9.81 |  | 84.9 + 9.81 |  |
| Control | 81.1 + 11.0 |  | 83.0 + 12.4 |  |
| ULTE4 (ng/mg)^d^ |  |  |  | -11% (-21%, 2%) |
| Intervention | 1.35 + 1.48 |  | 1.26 + 1.45 |  |
| Control | 1.41 + 1.55 |  | 1.43 + 1.50 |  |
| FeNO (ppb)^d^ |  |  |  | 7% (-19%, 40%) |
| Intervention | 14.6 + 2.25 |  | 15.3 + 2.55 |  |
| Control | 13.8 + 2.58 |  | 13.4 + 2.46 |  |
|  |  |  |  | **Dif. in mean (95% CI)** |
| Unscheduled clinical utilization, visits |  |  |  | -0.17 (-0.53, 0.19) |
| Intervention | - |  | 0.28 + 0.85 |  |
| Control | - |  | 0.42 + 0.67 |  |
| Steroid prescriptions, n |  |  |  | -0.07 (-0.67, 0.52) |
| Intervention | - |  | 0.59 + 1.24 |  |
| Control | - |  | 0.74 + 1.24 |  |
| **Dichotomous Outcomes** | **%** |  | **%** | **IRR (95% CI)** |
| C-ACT or ACT score < 19^a^, yes |  |  |  | 0.49 (0.23, 1.05) |
| Intervention | 18.2 |  | 8.6 |  |
| Control | 32.4 |  | 19.9 |  |
| Symptoms in past 2 wk, yes |  |  |  | 0.69 (0.46, 1.04) |
| Intervention | 54.6 |  | 38.9 |  |
| Control | 58.8 |  | 47.9 |  |
| ULTE_4_ (ng/mg) > median^b^, yes |  |  |  | 0.75 (0.52, 1.09) |
| Intervention | 46.3 |  | 38.6 |  |
| Control | 53.1 |  | 48.4 |  |
| Elevated FeNO, yes |  |  |  | 0.91 (0.51, 1.63) |
| Intervention | 40.6 |  | 31.0 |  |
| Control | 31.3 |  | 31.2 |  |
| **Discrete Outcome** | **Mean (SD)** |  | **Mean (SD)** | **IRR (95% CI)** |
| Symptoms in past 2 wk, days |  |  |  | 0.53 (0.29, 0.98) |
| Intervention | 1.76 + 1.97 |  | 1.32 + 2.37 |  |
| Control | 1.88 + 2.75 |  | 2.04 + 3.58 |  |
| Notes: Effect estimates reflect comparison of the change from baseline to the mid-study (6 months) and final (12 months) visits averaged, in the intervention versus control groups; estimates were calculated using generalized estimating equations in repeated measures linear models that included an interaction term for intervention group and 6 and 12 month follow-up visit values and were adjusted for baseline outcome values, age, sex, and season for all outcomes except for unscheduled clinical utilization and steroid prescriptions. For unscheduled clinical utilization and steroid prescriptions difference between groups was calculated using multivariate adjusted linear regression models; C-ACT: Childhood Asthma Control Test; ACT: Asthma Control Test; ULTE4: Creatinine-adjusted urinary leukotriene E4; FeNO: Fractional exhaled nitric oxide.  Footnotes: ^a^C-ACT or ACT score < 19 defines poorly controlled asthma;  ^b^UTLE4 median of 1.35 ng/mg creatinine;  ^c^Standardized ACT score is the total C-ACT or ACT score divided by the total score possible;  ^d^Standardized ACT score, ULTE4, and FeNO were log-transformed for analysis and, thus, the geometric mean for visits and the percent difference in mean change for the effect estimate are presented. | | | | |

| **Supplemental Table 4: Asthma health outcomes at baseline and post-randomization visits and effect of intervention on health outcomes post-randomization comparing intervention to control groups among HAPI Study adherent subset (N=68).** | | | | |
| --- | --- | --- | --- | --- |
| **Clinical Outcome** | **Baseline**  **Visit** |  | **6 and 12 Month Visits** | **Effect Estimate** |
| **Continuous Outcomes** | **Mean (SD)** |  | **Mean (SD)** | **Dif. in mean change (95% CI)** |
| Standardized ACT score^c, d^ |  |  |  | 11% (-11%, 39%) |
| Intervention | 82.7 + 10.0 |  | 84.8 + 9.65 |  |
| Control | 81.3 + 11.3 |  | 82.9 + 12.2 |  |
| ULTE4 (ng/mg)^d^ |  |  |  | -12% (-23%, 1%) |
| Intervention | 1.28 + 1.41 |  | 1.25 + 1.44 |  |
| Control | 1.40 + 1.52 |  | 1.42 + 1.50 |  |
| FeNO (ppb)^d^ |  |  |  | 7% (-20%, 42%) |
| Intervention | 15.5 + 2.38 |  | 16.2 + 2.51 |  |
| Control | 13.3 + 2.52 |  | 13.3 + 2.51 |  |
|  |  |  |  | **Dif. in mean (95% CI)** |
| Unscheduled clinical utilization, visits |  |  |  | -0.12 (-0.51, 0.28) |
| Intervention | - |  | 0.28 + 0.88 |  |
| Control | - |  | 0.38 + 0.65 |  |
| Steroid prescriptions, n |  |  |  | -0.03 (-0.64, 0.59) |
| Intervention | - |  | 0.59 + 1.27 |  |
| Control | - |  | 0.74 + 1.21 |  |
| **Dichotomous Outcomes** | **%** |  | **%** | **IRR (95% CI)** |
| C-ACT or ACT score < 19^a^, yes |  |  |  | 0.50 (0.23, 1.07) |
| Intervention | 19.4 |  | 9.2 |  |
| Control | 32.4 |  | 20.0 |  |
| Symptoms in past 2 wk, yes |  |  |  | 0.69 (0.44, 1.08) |
| Intervention | 58.1 |  | 38.5 |  |
| Control | 59.5 |  | 47.9 |  |
| ULTE_4_ (ng/mg) > median^b^, yes |  |  |  | 0.78 (0.50, 1.20) |
| Intervention | 40.0 |  | 36.2 |  |
| Control | 54.3 |  | 51.6 |  |
| Elevated FeNO, yes |  |  |  | 0.84 (0.45, 1.57) |
| Intervention | 46.7 |  | 31.3 |  |
| Control | 28.6 |  | 31.3 |  |
| **Discrete Outcome** | **Mean (SD)** |  | **Mean (SD)** | **IRR (95% CI)** |
| Symptoms in past 2 wk, days |  |  |  | 0.50 (0.25, 0.99) |
| Intervention | 1.74 + 1.97 |  | 1.35 + 0.30 |  |
| Control | 2.03 + 2.90 |  | 2.04 + 0.42 |  |
| Notes: Effect estimates reflect comparison of the change from baseline to the mid-study (6 months) and final (12 months) visits averaged, in the intervention versus control groups; estimates were calculated using generalized estimating equations in repeated measures linear models that included an interaction term for intervention group and 6 and 12 month follow-up visit values and were adjusted for baseline outcome values, age, sex, season, and controller medication use for all outcomes except for unscheduled clinical utilization and steroid prescriptions. For unscheduled clinical utilization and steroid prescriptions difference between groups was calculated using multivariate adjusted linear regression models; C-ACT: Childhood Asthma Control Test; ACT: Asthma Control Test; ULTE4: Creatinine-adjusted urinary leukotriene E4; FeNO: Fractional exhaled nitric oxide.  Footnotes: ^a^C-ACT or ACT score < 19 defines poorly controlled asthma;  ^b^UTLE4 median of 1.35 ng/mg creatinine;  ^c^Standardized ACT score is the total C-ACT or ACT score divided by the total score possible;  ^d^Standardized ACT score, ULTE4, and FeNO were log-transformed for analysis and, thus, the geometric mean for visits and the percent difference in mean change for the effect estimate are presented. | | | | |

| **Supplemental Table 5: Asthma health outcomes at baseline and post-randomization visits and effect of intervention on health outcomes post-randomization comparing intervention to control groups adjusted for Weatherization Program participation.** | | | | |
| --- | --- | --- | --- | --- |
| **Clinical Outcome** | **Baseline**  **Visit** |  | **6 and 12 Month Visits** | **Effect Estimate** |
| **Continuous Outcomes** | **Mean (SD)** |  | **Mean (SD)** | **Dif. in mean change (95% CI)** |
| Standardized ACT score^c, d^ |  |  |  | 9% (-14%, 37%) |
| Intervention | 83.4 + 9.53 |  | 85.0 + 9.63 |  |
| Control | 81.3 + 11.3 |  | 82.9 + 12.2 |  |
| ULTE4 (ng/mg)^d^ |  |  |  | -10% (-20%, 1%) |
| Intervention | 1.35 + 1.45 |  | 1.26 + 1.50 |  |
| Control | 1.40 + 1.52 |  | 1.40 + 1.44 |  |
| FeNO (ppb)^d^ |  |  |  | 3% (-21%, 33%) |
| Intervention | 15.5 + 2.3 |  | 15.8 + 1.11 |  |
| Control | 13.3 + 2.5 |  | 13.3 + 1.12 |  |
|  |  |  |  | **Dif. in mean (95% CI)** |
| Unscheduled clinical utilization, visits |  |  |  | -0.11 (-0.46, 0.23) |
| Intervention | - |  | 0.25 + 0.81 |  |
| Control | - |  | 0.38 + 0.65 |  |
| Steroid prescriptions, n |  |  |  | -0.08 (-0.65, 0.49) |
| Intervention | - |  | 0.53 + 1.18 |  |
| Control | - |  | 0.74 + 1.21 |  |
| **Dichotomous Outcomes** | **%** |  | **%** | **IRR (95% CI)** |
| C-ACT or ACT score < 19^a^, yes |  |  |  | 0.46 (0.22, 0.95) |
| Intervention | 15.8 |  | 8.3 |  |
| Control | 32.4 |  | 20.0 |  |
| Symptoms in past 2 wk, yes |  |  |  | 0.76 (0.52, 1.13) |
| Intervention | 55.3 |  | 38.9 |  |
| Control | 59.5 |  | 47.9 |  |
| ULTE_4_ (ng/mg) > median^b^, yes |  |  |  | 0.77 (0.53, 1.11) |
| Intervention | 46.0 |  | 37.5 |  |
| Control | 54.3 |  | 51.6 |  |
| Elevated FeNO, yes |  |  |  | 0.82 (0.46, 1.49) |
| Intervention | 43.2 |  | 31.0 |  |
| Control | 27.0 |  | 31.3 |  |
| **Discrete Outcome** | **Mean (SD)** |  | **Mean (SD)** | **IRR (95% CI)** |
| Symptoms in past 2 wk, days |  |  |  | 0.64 (0.36, 1.13) |
| Intervention | 1.63 + 1.88 |  | 1.43 + 2.53 |  |
| Control | 2.03 + 2.90 |  | 2.04 + 3.54 |  |
| Notes: Effect estimates reflect comparison of the change from baseline to the mid-study (6 months) and final (12 months) visits averaged, in the intervention versus control groups; estimates were calculated using generalized estimating equations in repeated measures linear models that included an interaction term for intervention group and 6 and 12 month follow-up visit values and were adjusted for baseline outcome values, age, sex, season, controller medication use, and Weatherization Program participation for all outcomes except for unscheduled clinical utilization and steroid prescriptions. For unscheduled clinical utilization and steroid prescriptions difference between groups was calculated using multivariate adjusted linear regression models; C-ACT: Childhood Asthma Control Test; ACT: Asthma Control Test; ULTE4: Creatinine-adjusted urinary leukotriene E4; FeNO: Fractional exhaled nitric oxide.  Footnotes: ^a^C-ACT or ACT score < 19 defines poorly controlled asthma;  ^b^UTLE4 median of 1.35 ng/mg creatinine;  ^c^Standardized ACT score is the total C-ACT or ACT score divided by the total score possible;  ^d^Standardized ACT score, ULTE4, and FeNO were log-transformed for analysis and, thus, the geometric mean for visits and the percent difference in mean change for the effect estimate are presented. | | | | |
